# New algorithms demonstrate untargeted detection of chemically meaningful changing units and formula assignment for HRMS data of polymeric mixtures in the open-source constellation web application

**DOI:** 10.1186/s13321-023-00680-5

**Published:** 2023-01-18

**Authors:** Dane R. Letourneau, Dennis D. August, Dietrich A. Volmer

**Affiliations:** Department of Chemistry, Humboldt University Berlin, 12489 Berlin, Germany

**Keywords:** Mass spectrometry, High resolution, Untargeted analysis, Software tool, Mass defect, Polymers

## Abstract

The field of high-resolution mass spectrometry (HRMS) and ancillary hyphenated techniques comprise a rapidly expanding and evolving area. As popularity of HRMS instruments grows, there is a concurrent need for tools and solutions to simplify and automate the processing of the large and complex datasets that result from these analyses. Constellation is one such of these tools, developed by our group over the last two years to perform unsupervised trend detection for repeating, polymeric units in HRMS data of complex mixtures such as natural organic matter, oil, or lignin. In this work, we develop two new unsupervised algorithms for finding chemically-meaningful changing units in HRMS data, and incorporate a molecular-formula-finding algorithm from the open-source CoreMS software package, both demonstrated here in the Constellation software environment. These algorithms are evaluated on a collection of open-source HRMS datasets containing polymeric analytes (PEG 400 and NIST standard reference material 1950, both metabolites in human plasma, as well as a swab extract containing polymers), and are able to successfully identify all known changing units in the data, including assigning the correct formulas. Through these new developments, we are excited to add to a growing body of open-source software specialized in extracting useful information from complex datasets without the high costs, technical knowledge, and processor-demand typically associated with such tools.

## Introduction

The last decade has seen the development of a plethora of creative and innovative open-source software tools for the analysis of mass spectrometry (MS) data [1, 2] Many of these tools serve to replace and/or augment data processing functions from proprietary instrument-manufacturer software packages, while others offer entirely new algorithms and automation techniques for tasks such as peak detection and calibration [3] assignment of molecular formulae [4, 5] unsupervised peak learning in mass spectrometry imaging data [6] comparison of tandem mass spectra [7] detection of repeating mass spectral features [8] statistical and multivariate analyses [9] in addition to a wide range of visualization, graphic and plotting tools [10, 12] Many of these algorithms and tools have been released as open-source software on platforms such as GitHub, bypassing both the high costs and limitations of proprietary, commercial software packages, such as those bundled with many mass spectrometers. In addition, users are free to modify, expand upon, and improve these tools, leading to potentially very exciting work in collaborative and interdisciplinary spaces.

High-resolution mass spectrometry (HRMS), in particular, allows for the probing of many features and parameters in a single experiment, while also generating increasingly complex and detailed datasets which can often require great time and effort by the researcher to extract the desired information [13, 14] With this in mind, algorithms for unsupervised mining of HRMS data have become increasingly important to obtain important information in reasonable timeframes, especially as HRMS continues to become a more affordable and accessible technique to researchers in a diversity of fields. Unsupervised MS data-mining techniques have been especially important in metabolomics fields [15–17] but have also been used to uncover hidden structures in mass spectrometry imaging (MSI) data [6] predict the structural similarity between two chemical structures based on their MS/MS fragmentation spectra [7] and investigate the authenticity of olive oil in combination with chemometric techniques [18] Machine learning algorithms have also been very useful in these types of analyses, assisting in the interpretation of complicated TOF–SIMS data of human hair [19] the creation of a risk warning system of chemical hazards in drinking water [20], and the detection of chemically adulterated urine. [21]

When performing high-resolution mass spectrometry on complex, heterogeneous samples such as those containing large, polymeric species (e.g, natural organic matter, oil samples, lignin), there are often patterns within the resulting spectra corresponding to the gain or loss of the repeating polymer unit. These patterns can be made more obvious visually by transforming the spectra into the mass defect space [22] In brief, although it has been covered extensively in many other publications [14, 23–26] the Kendrick mass defect is a data transformation where accurate *m/z* values are rescaled according to a known repeating “unit” in the dataset, and then plotted against the fractional mass (accurate subtracted from nominal mass) to produce a characteristic mass defect plot. In this “mass defect space”, compounds with identical mass defect can form horizontal series, representing the loss or gain of the repeating “unit” of transformation. In complex spectra containing multiple polymeric analytes, the patterns which can result from this transformation and re-plotting can be very complex, and the plot generated from transformation by just one “base” unit can contain horizontal trends based on many different changing units.

Needless to say, the manual labor involved in tracing these mass defect patterns and extracting the desired information can be difficult, especially in a spectrum containing thousands to hundreds of thousands of peaks. Several automated detection techniques have been proposed. Loos and Singer developed a non-targeted algorithm to perform homologue series extraction from LC-HRMS data [27] The algorithm was evaluated on ten effluent samples from Swiss sewage treatment plants, and was able to identify series of known homologues, as well as numerous nontargeted peak series. Verkh et al. developed custom scripts, written in R, to observe DBE-O, mass and intensity shifts in LC-HRMS spectral features [28] This included the identification of CH_2_ and C_2_H_2_O series in KMD-transformed data. Bugsel and Zwiener created a MatLab script to automatically detect poly- and perfluoroalkyl substances in LC-HRMS spectra of contaminated soil samples, successfully identifying CF_2_, CF_2_O, and C_2_F_4_O repeating units [29] Along similar lines, our group has developed Constellation, an open-source web application which can perform unsupervised detection of linked series in mass-defect transformed HRMS data [30] The initial version of the software offered a variety of innovative tools for manipulating HRMS data in the mass defect space, as well as an unsupervised trend finding algorithm which was able find linked series in the mass defect space.

**Fig. 1:**
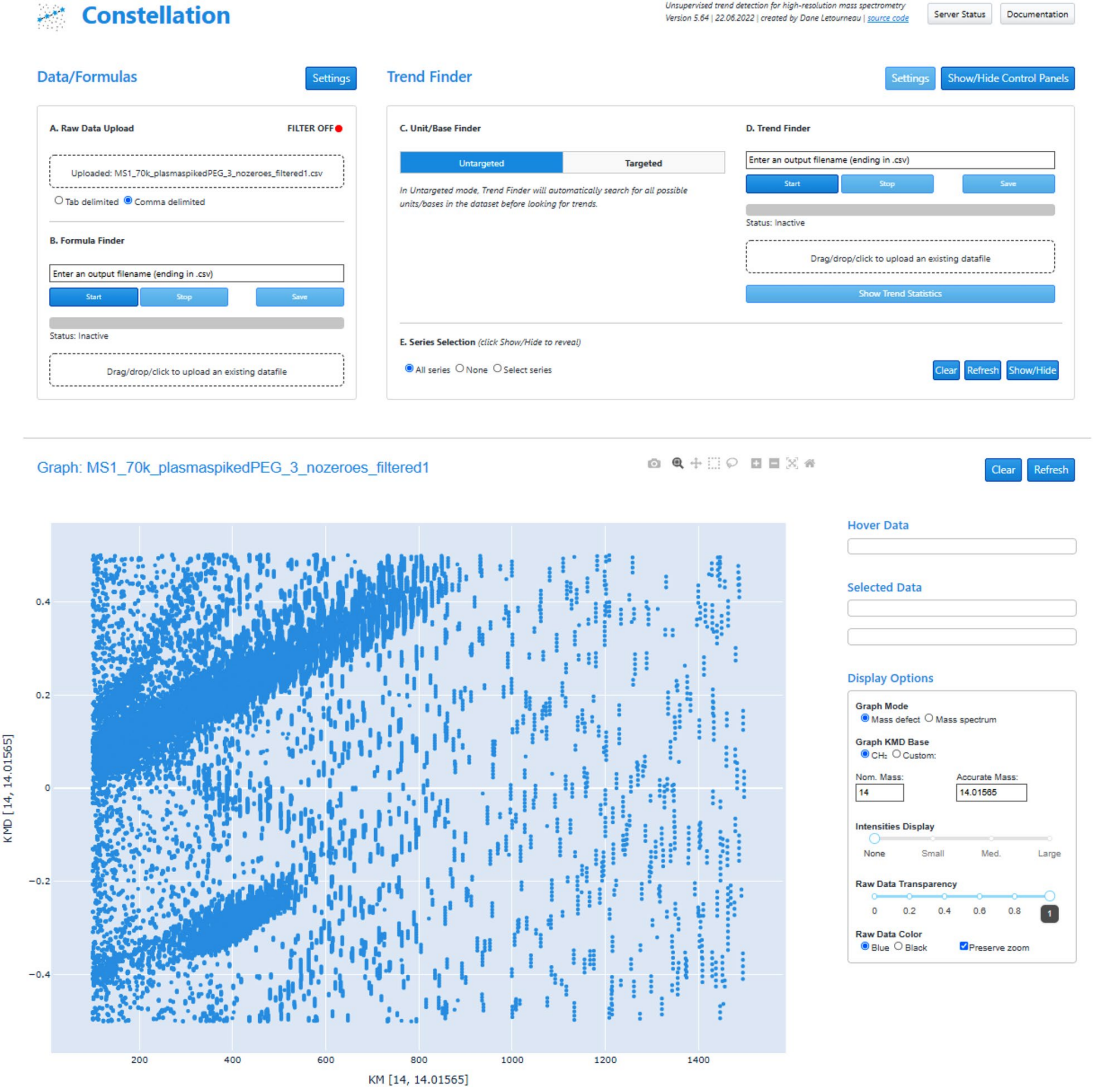
Constellation web-application interface (as of the time of publication). Shown is the plasma-spiked PEG dataset from da Silva et al. [8] loaded into the application, displayed in mass-defect mode in the Graph area

Constellation has been in continual development since its initial publication and release, with a particular focus on incorporating more options to directly assign chemical meaning (i.e., molecular formula information) to both raw MS signals and polymeric changing units. Initially, the software did not offer any built-in molecular formula finding capabilities; here, an algorithm incorporated from the open-source CoreMS software package [11] allows users to assign formula information for ions in the HRMS dataset. Following from this, two newly designed “unit/base finding” algorithms assign formula information for any changing units discovered in the dataset. These algorithms are then successfully evaluated on a collection of open-source HRMS datasets containing polymeric analytes (PEG 400 and NIST standard reference material 1950, both metabolites in human plasma, as well as a swab extract containing polymers) [8] and are able to successfully identify all known changing units in the data, including assigning the correct molecular formulas. All algorithms are demonstrated and evaluated within the open-source Constellation software environment, accessible easily via any web browser [31] Fig. 1 displays a screenshot of the current Constellation interface as of the time of publication.

## Implementation

### Constellation program structure

Constellation consists of two parts: A web interface, based on the open-source Dash architecture (Plotly Inc.) [32] enables users to upload a raw HRMS data file, manipulate their data within the mass defect space using the built-in graph, and send requests to our back-end server for data processing tasks such as unit/base finding, unsupervised trend detection or formula finding routines. The back-end server cluster, located at Humboldt University Berlin, receives these requests via the Celery distributed task queue [33] performs the computationally-intensive processing (so the end user’s computer is not responsible for the workload), and sends the results back to the web interface. To enable these functionalities, Constellation has a large number of dependencies, viewable in several requirements files stored in the Constellation GitLab repository [34].

Both the front- and back-end components of the software are designed to be run as system services on dedicated servers and require the simultaneous operation of two instances of Celery and one instance of Redis, also running as system services. The first instance of Celery, here called “frontcelery”, runs on the web server hosting the front-end web interface, and functions to store information via Redis when making requests to tasks running on the back-end server via a second Celery instance called “backcelery”. This is necessary, as Dash applications are structured around several “callbacks” which take in information from the web application interface, process it, and return some sort of output [32] Callbacks are based on HTTP requests and will therefore likely timeout if a callback takes too long to complete. As we have several callbacks making requests to the back-end task architecture (which, depending on the task, can take minutes to even hours to complete), we used a newer Dash feature called “Long Callbacks” [35] which relies on Celery and Redis to enable longer timeouts in certain situations. This is the primary function of “frontcelery”, in addition to some small maintenance tasks performed by a scheduled queue called “celerybeatfront”.

“backcelery”, on the other hand, is an instance of Celery functioning to manage the computationally demanding tasks we have written in “serverapp.py”, which runs on a dedicated server at Humboldt University Berlin. Requests are received via HTTP from the front-end web application via the built-in routing features in Flask [36]. The routing functions on the back-end server then collect the incoming data (in JSON format) and call the relevant Celery task asynchronously, before sending back a task ID to the front-end so the user can receive progress and task update information. It should be noted that we have designed two types of tasks here: “grouped” and “individual”. Individual tasks contain all the code needed to process incoming information within one Celery task, and send back updates via the self.update_state () function. Grouped tasks, on the other hand, attempt to distribute processor load for certain functions by splitting up the data and sending pieces to potentially hundreds to thousands of individual sub-tasks. These are slightly more complicated to monitor, and instead of receiving text-based updates from each sub-task, we simply count how many of these sub-tasks have been marked as completed and report this number to the front-end. In addition, upon completion, the code in the front-end callback has to re-assemble the information coming back from all grouped sub-tasks and evaluate if all information is present before proceeding to the next step. Finally, like “frontcelery”, “backcelery” also performs some small scheduled maintenance tasks via a queue called “celerybeatback”.

A note on data security: Constellation ensures sensitive user data is never compromised by implementing secure HTTP connections (HTTPS) to both the web interface and the remote data processing server. Data uploaded to (or generated by) Constellation is stored temporarily on our server until the user is finished their session, after which the data are deleted. An anonymous ID, randomly generated for each user (or each browser tab or window that a user opens), is the only information retained by Constellation, and only as a way of tracking software usage.

### Increasing file-size limitations when uploading and generating data

The original version of Constellation [30] limited raw MS file uploads to a maximum of 5000 data points (~ 200 kb of information). This was due to the use of data structures (in particular, the Dash core component “Store” [37]) which relied on the end user’s web browser to store both uploaded MS data and data generated by the software itself (i.e., returning series information from trend finding). Given that typical HRMS datasets are quite large, this was a high priority area for further development and improvement. Since the software was published, the file upload components in the front-end “webapp” have been upgraded to the open-source Dash Uploader [38] and the back-end “serverapp” has been migrated to a dedicated rack server run by our group and located in the Institute of Chemistry at Humboldt University Berlin (funded by the Berlin University Alliance BUA 501_LinkLab grant). This finally allowed for the increase of file-size limitations to 10 mb for both raw MS file uploads and for the storage of larger datasets resulting from trend-finding and formula-finding activities, via secure temporary files saved directly to our servers. This represents a significant improvement in the abilities of the Constellation web application so far, with even less demand placed on the end user’s computer or Internet browser.

### Integration of molecular formula finding algorithm from corems

The molecular formula finding function from CoreMS [11] was integrated according to the package’s documentation. This required setting up a Docker container running as a system service to give the function access to an SQL database of potential molecular formulas. A Celery task called “formulafinder” was created in the back-end “serverapp” which first sets CoreMS molecular search settings according to user input parameters (or a set of defaults if they have not been modified), creates a “mass spectrum object”, and then calls the *SearchMolecularFormulas* class to perform the database search. A simple interface was built and added to Constellation, allowing users to adjust settings and to monitor the formula-finding calculations, as well as save the results as a.csv file to their computer.

### Development and integration of new unit/base finding algorithms

All algorithms developed for Constellation in this work are scripts written in Python and utilize several common Python packages such as Pandas [39] and Numpy [40] for data structuring and comparison. Scripts were debugged first by running through the Python interpreter (version 3.6) locally, and then migrated to our test (“beta”) high-powered data processing server via a development branch in our Git repository for debugging within our task management framework, controlled by the open-source Celery distributed task queue [33] Celery allows us to manage distribution of task workload across our server’s 112 CPU cores, while also allowing for separation of task queues for each user if there are multiple users operating the software simultaneously. We also use Flower, a “web based tool for monitoring and administrating Celery clusters” [41] to check for any exceptions or errors encountered in Celery tasks. The entirety of the new unit/base finder algorithms, including loading of formulas and evaluation of units within the raw HRMS dataset, takes place in a single Celery task called “unitbasefinder”. We attempted, at first, to split up the workload among multiple tasks in a Celery structure called a “group”, however it was unsuccessful due to the size of the data being loaded into each task. With some optimization, however, this task-splitting may be possible in the future and enable significant speed improvements for the unit/base finding task.

To be considered ready for production, the Celery task running the algorithms had to run free of errors or exceptions, deliver updates on calculation progress to the front-end “webapp” at regular intervals and shut down cleanly both in the case of an early shut-down event initiated by the user or in the usual case of the task completing and returning results. Once the algorithms had been sufficiently debugged on the testing (“beta”) server, they were pushed to our production (“alpha”) server in the master branch of our GitLab repository [34] The “alpha” and “beta” servers are clones of each other and we observed a successful deployment to the “alpha” server after testing on the “beta” server at every stage of development. It should be noted that every new feature added to Constellation in this work (e.g., increasing file-size limitations, integration of the CoreMS molecular formula search) and any future developments will follow this same path from development to production, so that the Git master branch in the repository will always reflect the current production version of Constellation.

## Results and discussion

### Molecular formula finding algorithm

Constellation originally allowed users to upload an output file from the FormulaAssignment script, part of the Python-based FTMS Visualization software package [10]. This data layer was matched with a user-uploaded raw HRMS dataset, so that in Constellation’s graphing interface, points that were hovered over or selected would display a molecular formula annotation (if available). However, there was a desire to incorporate a formula assignment routine directly into the Constellation interface. Since the publication of FTMS Visualization, there have been a number of other significant software packages developed which offer improved formula finding capabilities as well as various other features for processing and manipulating HRMS data.^4^, In view of this, and wanting to work with a software package in active development, we chose to use CoreMS, [5], 9, 11, 12, 42a new set of HRMS data tools from Pacific Northwest National Laboratories in Richland, WA, USA.

CoreMS aims to be a “comprehensive mass spectrometry framework for software development and data analysis of small molecules analysis,” [11] and offers an impressive selection of tools for loading raw HRMS data from proprietary vendor formats, signal processing for FT-MS (apodization, zero-filling, etc.), baseline subtraction and smoothing, recalibration routines, and of interest to us, molecular formulae search and assignment routines. These functions are all easily accessible through the usual methods of installing and importing Python packages, with the exception that the molecular formula finding scripts require the user to set up and run a Docker-based SQL molecular formula database. In the case of Constellation, this had to be run as a system-service on our high-performance server.

CoreMS was installed on our server and implemented in the Constellation GUI via a small graphical interface to give users access to these molecular formula-finding routines. If a valid raw HRMS dataset has been loaded into the program, users can simply click “Start” to run the formula finding routine on the remote data-processing server (assuming the default parameters are sufficient), wait for a result, and download the resulting.csv output file to their computer. In the case that they want to adjust any parameters from the defaults, a “Settings” window is available with all parameters from the CoreMS script mapped to input fields, enabling fine-tuning of errors, double-bond equivalents (DBE), isotopologue and minimum peaks filters, and elemental limits.

## New algorithms for generating mass defect units/bases

### Overview

In our previous publication detailing an earlier version of Constellation [30] we have described how the unsupervised Trend Finder algorithm at the heart of the software first generated a list of potential units/bases to test based on frequently occurring “gaps”, or distances between *m/z* values, in the raw HRMS dataset. Until now, this approach resulted in very large list of floating-point numbers, with no chemical meaning necessarily reflected in these patterns. As both a way to narrow this list down (saving a significant amount of time in the unsupervised trend search), and to offer a starting point for the interpretation of the trend finding results, we have developed two new unit/base finding algorithms which search for only *chemically meaningful* repetition patterns in the dataset; that is, only units of change which can be assigned a reasonable chemical formula.

**Fig. 2:**
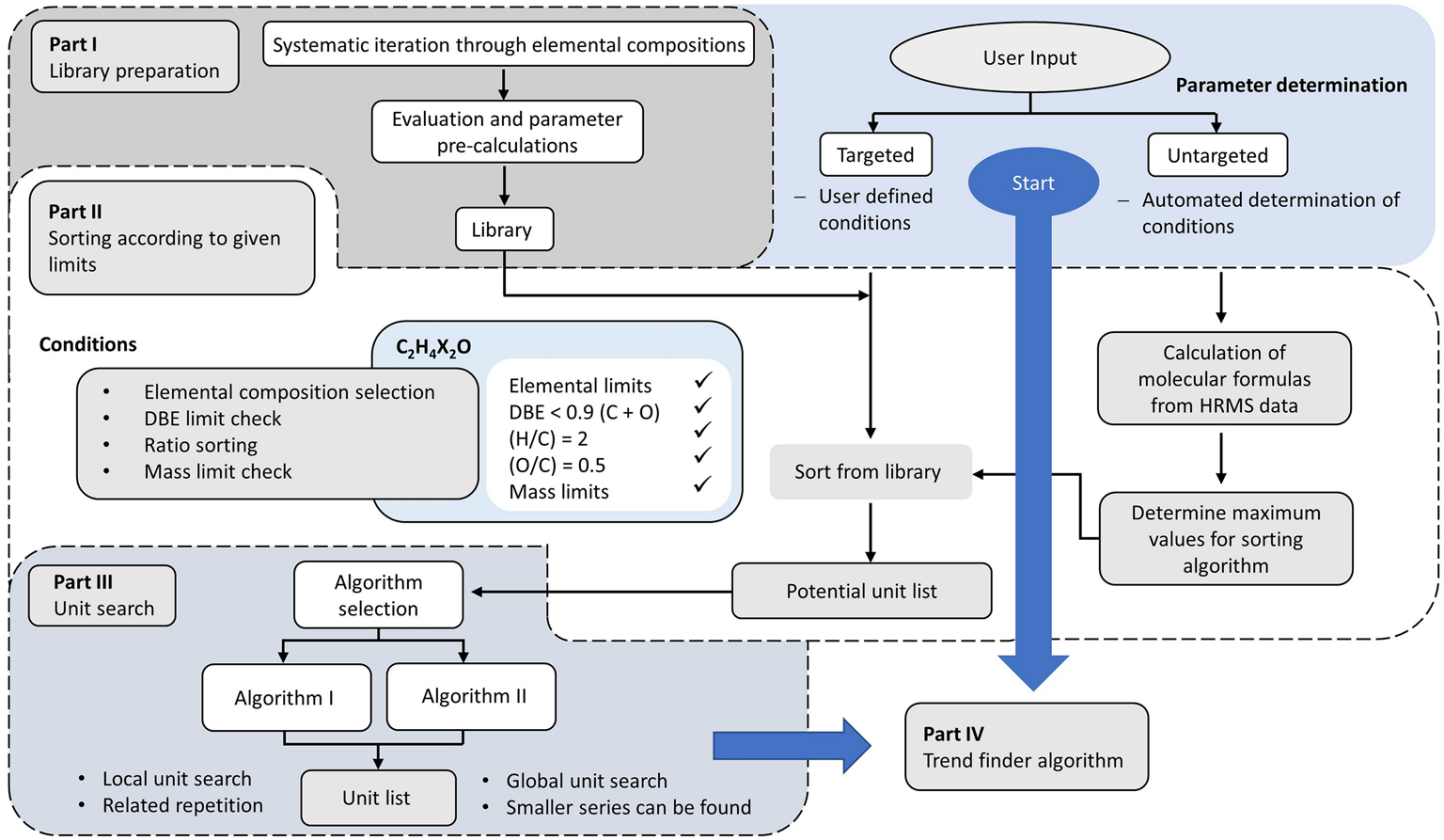
Workflow chart for unit/base finding processes in Constellation

These algorithms can operate in either “untargeted” mode (where all settings are either optimized by Constellation based on the input MS dataset or set to “reasonable” defaults based on the datasets we used in testing the software), or “targeted” mode (where the user can fully customize all settings). They function by loading a pre-generated formula library, from which potential units/bases are selected according to parameters including elemental limits and minimum/maximum size limits. The raw MS dataset is then searched to see if any of these potential units/bases are present, and if so, at what frequency they repeat. The resulting list of units/bases is then either directly sent to the Trend Finder algorithm (in “untargeted” mode) or displayed in a selection box for the user to curate as they like before trend finding (in “targeted” mode). Figure 2 displays the workflow when using Constellation with the new unit/base finding algorithms.

### Library generation

The new unit/base finding algorithms require a library of potential starting formulas, generated by a loop iteration through the elements C, H, S, O, N, P, F, Cl, Br, Si and X according to elemental limits. We introduce X to represent a connecting point from the changing polymeric unit to the molecular scaffold. All elements in the iteration are labeled with their valence ν, which is needed for the evaluation of the molecular formula according to the double-bond equivalent (DBE) [43, 44] The DBE relies on chemical rules and can be applied as a constraint to evaluate elemental compositions [43] – as an example, the Lewis and Senior rules can be used as another chemical based validation approach [45–47]. If a non-integer or negative DBE value is obtained, the initial molecular formula for the potential unit/base was incorrect [43]. The DBE is calculated as

1 $$\mathrm{DBE}=\frac{1}{2}\sum [{E}_{i}({\nu }_{i}-2)]+ 1$$

in which *E*_*i*_ is the number of atoms of the element *i*. [43] However, Eq. (1) has some drawbacks in our iteration approach since it would accept unreasonable structures like NX, which are generated in our systematic iteration. Therefore, we derived Eq. (2) for the validation of our formulas, which also relies on fundamental valence principles and can be seen as a related but restricted approach for the DBE calculation.

2 $$\frac{1}{2}\sum {E}_{i,\nu \ge 2}{\nu }_{i}-\frac{1}{2}\sum {E}_{i,\nu =1}-\chi (\sum {E}_{i,\nu \ge 2}+\mathrm{DBE}-1)=0$$

with $\chi$ defined as

3 $$\chi =\left\{\begin{array}{c}1, \sum {E}_{i,\nu \ge 2}>1\\ 0, \sum {E}_{i,\nu \ge 2}=1\end{array}\right.$$

Some elements such as sulfur and phosphorus can have different valence states in organic compounds, hence different valence combinations must be considered. Therefore, each valence state of an element is independently included in the iteration. Our expression for the DBE calculation in conjunction with the pre-defined valency includes molecular formulae which comprise multiple elements at higher valence states as well as mixed combinations of valence states. These molecular formulae might be excluded if only normal valences are considered due to negative DBE values. Table 1 lists the valence states and compositional limits used for library generation.

**Table 1:** Valence states and default compositional limits used for library generation

Element	C	H	S	O	N	P	F	Cl	Br	Si	X
Valence (ν)	4	1	2, 4, 6	2	3	3, 5	1	1	1	4	1
Default limits	0–10	0–20	0–4	0–4	0–2	0–2	0–4	0–2	0–2	0–1	1–2

### Formula selection

The unit/base finding process begins with the selection of potential formulae from the main library. Our generated library covers millions of molecular formulae, and it is therefore ineffective to apply the whole library in our search algorithm due to high computing time and the possibility of false assignments. Hence, the potential formulae are screened according to elemental limits, the maximum DBE rule [48], elemental ratios [47] (0.3 ≤ H/C ≤ 4.0, 0 ≤ N/C ≤ 1.3, 0 ≤ O/C ≤ 1.2, 0 ≤ P/C ≤ 0.3, 0 ≤ S/C ≤ 0.8, 0 ≤ F/C ≤ 1.5, 0 ≤ Cl/C ≤ 0.8, 0 ≤ Br/C ≤ 0.8, 0 ≤ Si/C ≤ 0.5) and the mass limits *m*_*Unit*_ (14 ≤ m ≤ 200 Da). All of these parameters and conditions can be changed/disabled by the user in “targeted” mode or left to their defaults in “untargeted” mode. The final evaluated list of formulae is then saved and sent to the unit/base finding algorithms.

### Algorithm selection and data filtering

After selecting potential formulae, we have to choose which of the two different unit/base finding algorithms to use, since the calculation time scales differently with the size of the data and unit/base set for each algorithm. In “untargeted” mode, algorithm II is chosen as a default based on better performance for large formula libraries and input MS datasets (described in the “Scaling of algorithms” section below). However, this is not the only factor to consider when choosing, as each algorithm can have advantages and disadvantages in certain circumstances, as will be discussed in the following sections. Therefore, in “targeted mode”, the user can choose which algorithm is employed, or test both individually. It is important to note that in some cases and with certain custom settings, this may dramatically increase analysis time.

This can be mitigated, if desired, by employing Constellation’s built-in Data Filter, which gives the user two options to reduce the size of their dataset. The “Target data length” option filters by selecting the *x* number of most intense peaks in the raw MS dataset while excluding the rest. Setting *x* to a value lower than the length of the uploaded raw MS dataset therefore removes less intense peaks to leave the user with a smaller dataset. The “Intensity threshold” option filters by removing all peaks below a specified minimum intensity threshold *i* (in %). In this case, the intensities of all peaks are normalized to the highest-intensity peak and then normalized intensities smaller than *i* are removed. It should be noted that this filtering can end up excluding information from the raw MS dataset which may be important to the user, so it must be used with discretion. It is recommended to try data filtering when evaluating the unit/base and trend finding algorithms to shorten the analysis time, after which parameters can be adjusted accordingly, and the analysis re-attempted with the full-length dataset once there is some time to wait for a result.

### Algorithm I – local search

**Fig. 3:**
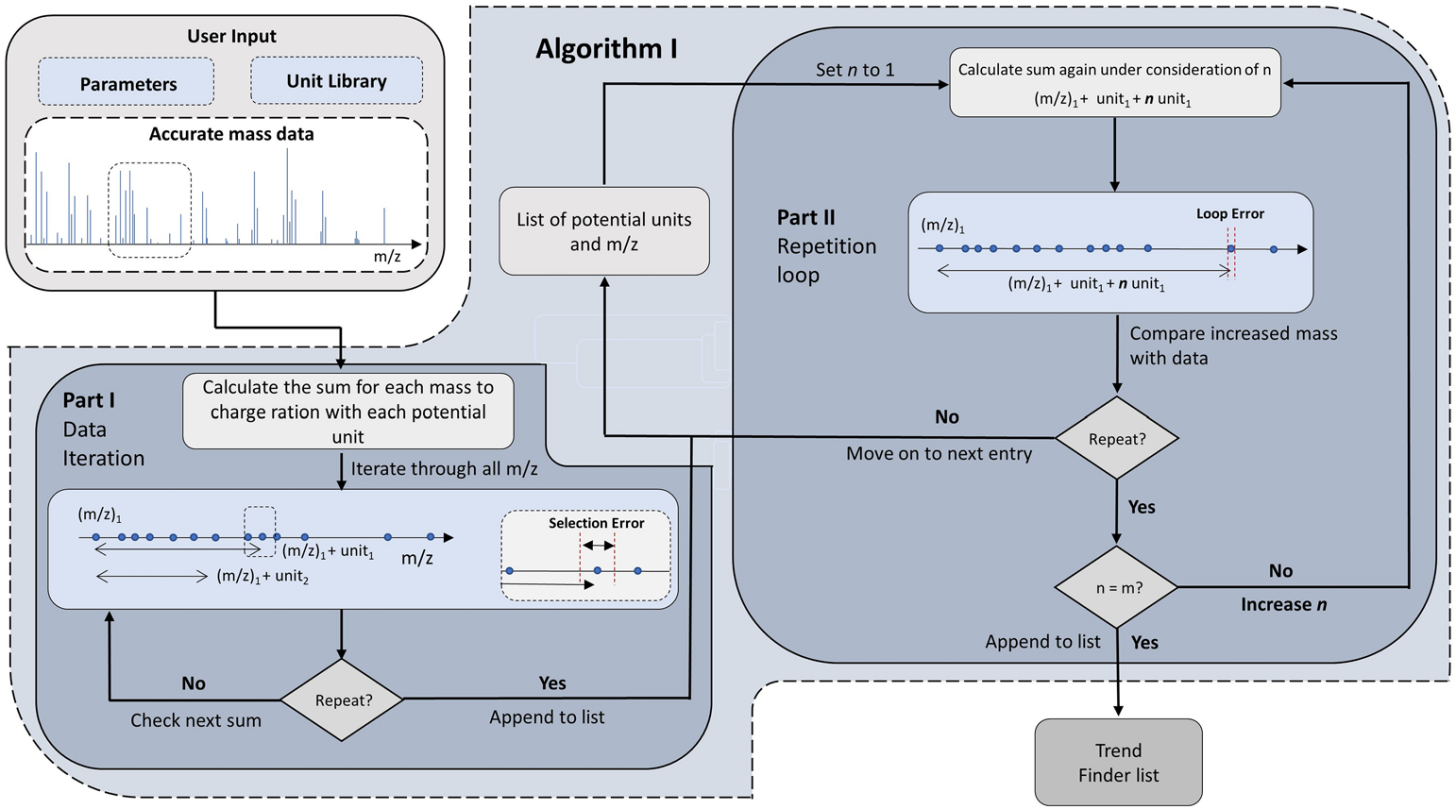
Flowchart for Unit/Base Finder algorithm I

The first unit/base finder algorithm (see the flowchart in Fig. 3) adds the accurate mass of each unit in our formula list to the *m/z* value of each data point in the raw HRMS data set to generate a new dataset, which is then compared to the original HRMS dataset (± selection error). If any points between the two datasets match, the successful unit and the corresponding sum of the accurate mass plus the unit are saved to our output list. The algorithm then takes each sum in this output list, adds the unit again to the sum and searches the initial HRMS data set for matches (± loop error). The last step is repeated in a loop, where for each successful iteration, we increase the repetition counter *n* by one until it is equal to the number of search steps *m.* The parameter *m* can be chosen by the user and has a strong impact on the computational time and the length of the unit list, especially if high error ranges are defined. If *m* is reached, the unit is saved to be passed on to Trend Finder.

Each loop iteration represents the addition of another equivalent of the changing unit to a fixed accurate mass from the dataset. Therefore, the first algorithm identifies repetitions of a changing unit in sequence (“locally”), which means the unit will have a high probability of yielding a series in Trend Finder. This “local search” approach generates a small, curated list of units/bases and is best suited for highly complex datasets, reducing the number of potential units/bases compared to the “global search” approach of algorithm II. Therefore, for complicated datasets, algorithm I can strongly decrease the overall computing time when running Trend Finder in “untargeted” mode.

### Algorithm II – global search

**Fig. 4:**
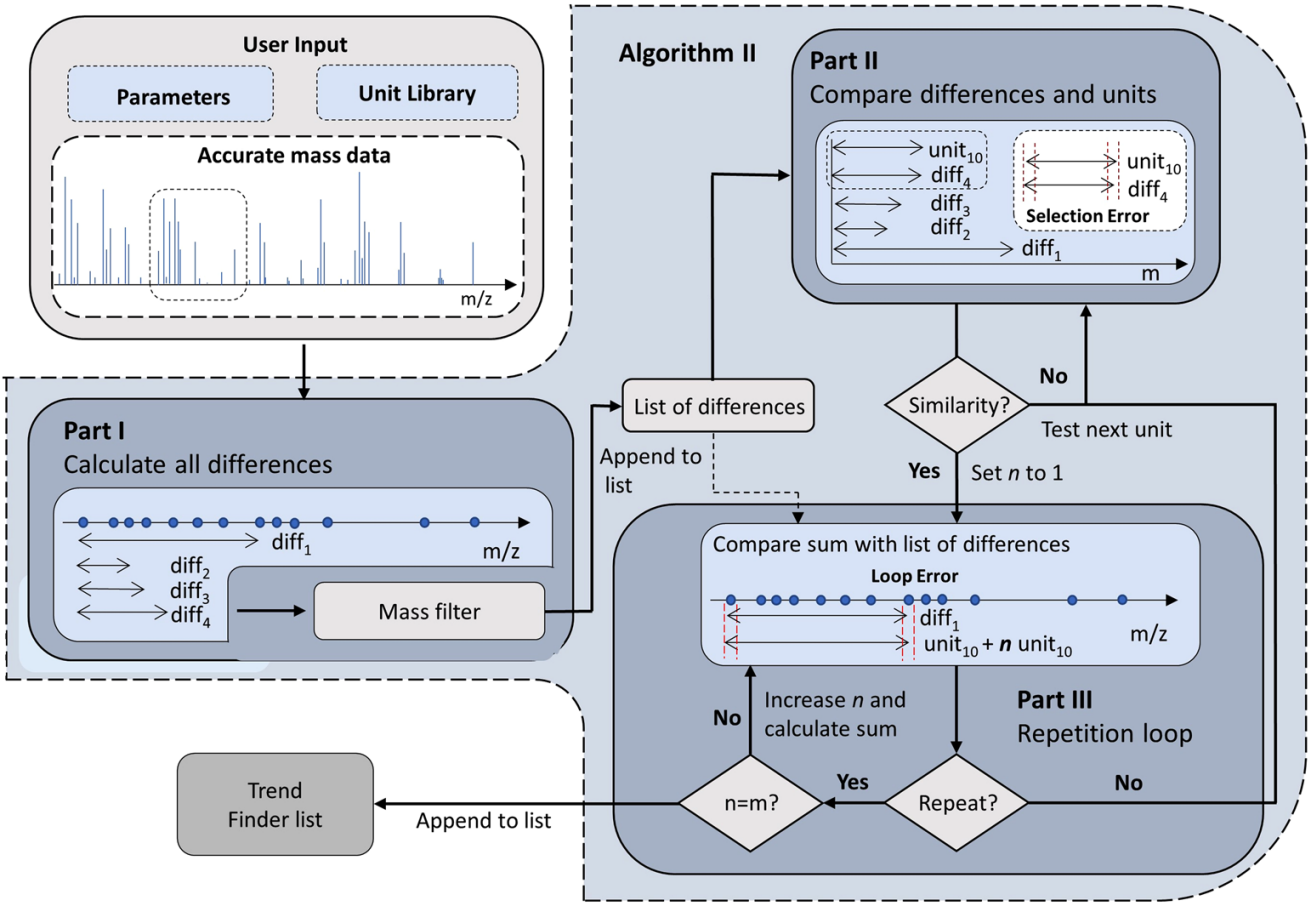
Flowchart for Unit/Base Finder algorithm II

The second unit/base finder algorithm (see the flowchart in Fig. 4) calculates the distances between each data point in the HRMS dataset to gain a list of differences. This list is compared to the list of potential units in the formula library (± selection error). If there is a match, the corresponding unit is taken and multiplied by a repetition counter *n,* and then this value is compared again to the list of differences (± loop error). This step is repeated, and for each successful loop iteration *n* is increased by one until it is equal to the selected number of search steps *m*. If *m* is reached, the unit is saved to be passed on to Trend Finder. This approach compares all values in the list of differences to each unit in the library, and does not apply any restrictions on the local environment of a found pattern (i.e., a changing unit does not have to repeat in sequence) – therefore, the results correspond to a “global search” within the raw MS dataset.

The advantage of the second algorithm is a high sensitivity for patterns which correspond to changing units showing minimal sequential (or “local”) repetition. Moreover, this approach shows better scaling when using larger libraries of potential starting formulas or larger raw MS datasets as compared to the local search approach in algorithm I (see “Scaling of algorithms” section below for a detailed breakdown of this scaling). Therefore, algorithm II is suitable for fast analysis of very large HRMS data sets. However, it does not consider if the found repetitions are related to each other (i.e., discovered sequentially in the dataset), which may result in a lower probability of these units/bases returning series in Trend Finder. There are also usually more units/bases returned from this algorithm, potentially resulting in a longer analysis time when trend finding in “untargeted” mode.

### Algorithm parameters

Both algorithms depend on numerous parameters, as can be seen in Figs. 3, 4. All parameters can be customized by the user in “targeted” mode or decided automatically by Constellation in “untargeted” mode, without any pre-optimization or understanding of the algorithms. In “untargeted” mode, “reasonable” defaults are assumed for most parameters, which were optimized in development for five HRMS datasets containing polymeric species from various sources. In this mode, Constellation is also able to make some automated parameter adjustments based on the input raw HRMS dataset – for example, the maximum size of a potential changing unit will be set to the largest *m/z* value in the dataset divided by the minimum number of desired repetitions.

### Evaluation of algorithms

To check that both new unit/base finding algorithms were functioning according to expectations, we tested them on multiple HRMS datasets of polymers with known repeating units. These datasets were obtained from the MassIVE database, a resource developed by the Center for Computational Mass Spectrometry at the University of California, San Diego, USA to “promote the global, free exchange of mass spectrometry data.” [49] The datasets correspond to a published study by da Silva et al., who developed a computational method for removing repeating mass spectral features [8] In the study, the authors applied their method to mass spectra of PEG 400 and NIST standard reference material 1950 (metabolites in human plasma) spiked with PEG 400, as well as a swab extract containing various polymers. Given that some of the repeating signals here are “knowns”, and the datasets from the study are publicly available, it offered an opportunity to evaluate whether our newly developed algorithms were able to detect these previously identified changing units. Both algorithms were tested in “untargeted” mode (where settings were chosen automatically by the software or set to “reasonable” defaults) and “targeted” modes (where various settings were customized to help find the changing units of interest).

To start, we applied both algorithms to search for repeating units within the “contaminants” category, defined by da Silva et al. as containing PEG 400 (repeating unit of C_2_H_4_O), perfluorinated molecules (repeating unit of CF_2_) and PPG (repeating unit of C_3_H_6_O) [8] We were able to successfully find the PEG repeating unit in the “plasma sample spiked with a swab” dataset with both algorithms in “untargeted” mode. The CF_2_ unit was also found by both algorithms, but due to a weak local repetition, only the global search approach (algorithm II) was successful in “untargeted” mode, while the local search approach (algorithm I) was able to find the CF_2_ unit in “targeted” mode with optimized parameters. It should also be noted that the PPG unit was found by algorithm II in “untargeted” mode, despite the referenced study not finding any repetitions of this unit in the dataset [8] We also investigated the ability of our algorithms to search for the CH_2_, C_2_H_4_, C_3_H_6_ and C_4_H_8_ repeating units from the “composition” category defined by da Silva et al. [8] We did not expect a highly defined local repetition pattern for these units, therefore algorithm II was predicted to yield better results as compared to algorithm I. As expected, in “untargeted” mode we were able to find all known repeating units with algorithm II, while algorithm I only returned a partial list. However, in “targeted” mode with some adjustment of parameters, we were able to find all units with both algorithms.

**Fig. 5:**
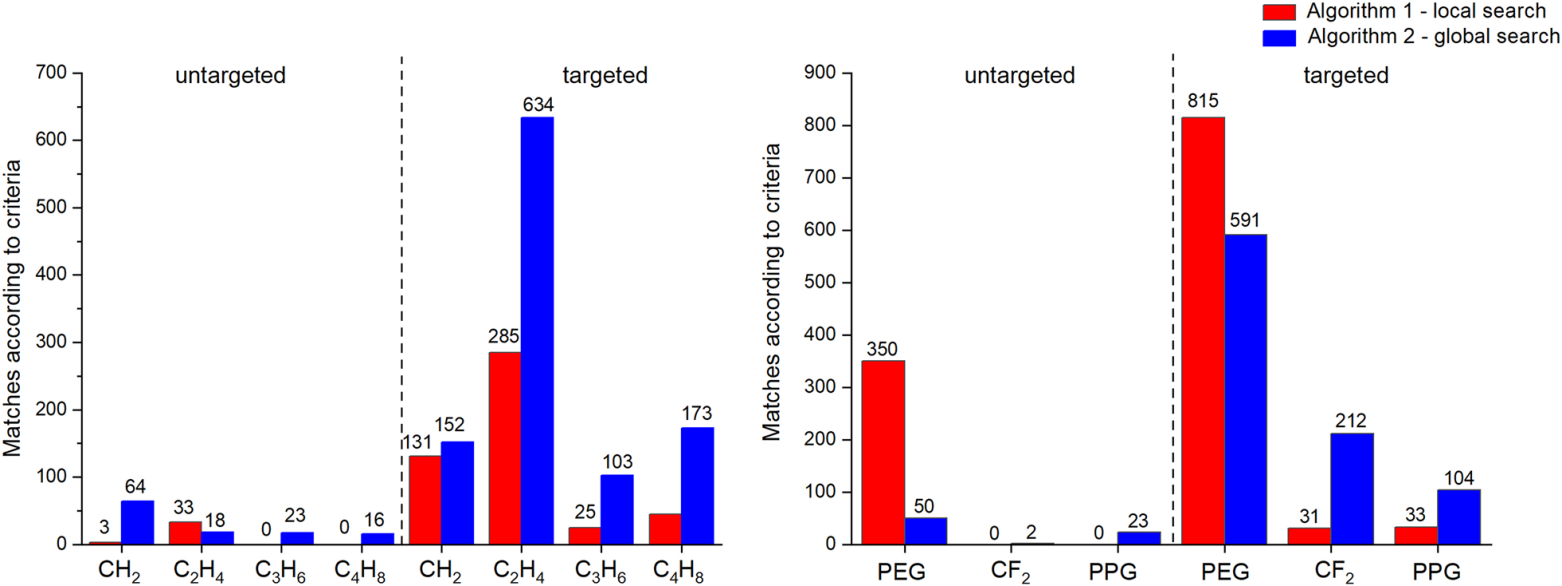
Evaluation results for both algorithms (targeted and untargeted modes) for the “plasma sample spiked with a swab” dataset from da Silva et al.,[8] filtered to 988 data points using the “Target data length” option in Constellation’s Data Filter. Each changing unit reported in the previous study (abscissa) is shown along with how many times it was detected in the dataset using the new unit/base finding algorithms developed here (ordinate)

These evaluation steps showed that our algorithms were able to detect all previously identified changing units in these datasets, including an additional unit not originally identified in the referenced study. Given their ability to correctly find the expected values, we expect the algorithms will function in a similar manner to detect new, unknown changing units in untargeted analyses, and hopefully provide added value to the analyst when looking through complex HRMS datasets for repeating patterns in an automated, unsupervised fashion. In “untargeted” mode, where parameters are automatically chosen or set to defaults by Constellation, algorithm II was consistently able to identify all the expected changing units in our evaluation, so we chose it as the default algorithm in this mode. Algorithm II is also the default in “targeted” mode, although here the user can of course switch to algorithm I and adjust other parameters if desired. Figure 5 summarizes these evaluation results visually, including the comparison of “targeted” and “untargeted” modes with both algorithms.

### Scaling of algorithms

The impact of the size of both the raw MS dataset and library of potential starting formulas on the performance of both algorithms was investigated. We took the publicly available “plasma sample spiked with a swab” dataset from da Silva et al. [8] and filtered it multiple times to generate four new datasets containing the top 1000, 2000, 3000, or 4000 peaks from the original dataset based on signal intensity. Multiple formula libraries were then generated with a library creation script (the same used to create the main library for the unit/base finding algorithms, as described earlier). The parameters for formula library generation were changed each time to give 9 libraries varying in size from 127 to 951725 potential changing-unit formulae.

**Fig. 6:**
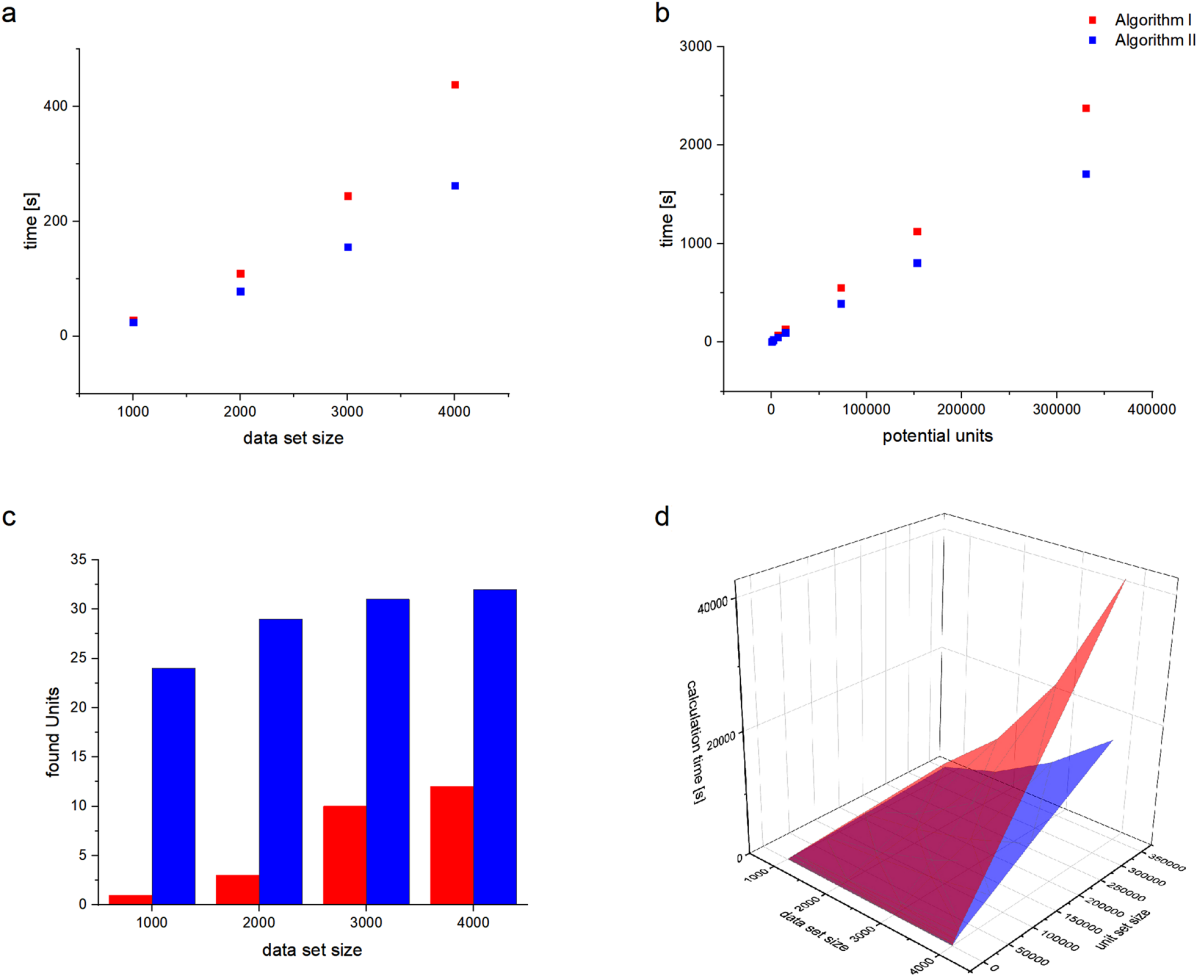
Algorithm scaling, **a **with different data set size, **b **with increasing length of the potential unit lists; both indicate a better performance for algorithm II. **c **Comparison between the number of units found by both algorithms with a constant unit list (127 entries) and increasing data set size, showing a smaller number of units for algorithm I due to consideration of local repetition, **d **3D plot of calculation time in dependency of data set size and unit list length

These libraries were then used in unit/base finding for each of the filtered datasets, with both algorithms and default settings for all parameters, while recording computing time and number of found units. The results (displayed in Fig. 6) show that the performance for larger unit libraries and raw MS datasets was better for the global search approach (algorithm II), but for smaller datasets–independent of the size of the used library – the local approach (algorithm I) demonstrated better performance. Moreover, algorithm I yielded a smaller list of units, as was expected given that algorithm II does not require units to repeat “locally” (in sequence). This trend was independent of the size of the chosen formula library. These results helped in setting reasonable defaults for unit/base finding tasks and should also help inform users as to what options may give the best performance for their dataset when customizing parameters to their liking.

## Conclusions

In this study, we introduced several new algorithms for assigning chemical meaning to both raw MS peaks and potential polymeric changing-units in HRMS datasets of complex samples, demonstrated here within the open-source Constellation software environment. Firstly, a formula assignment interface gives users access to the molecular formula finding algorithm developed by Dr. William Kew and Dr. Yuri Corilo as a part of their CoreMS package, running live on a server cluster at Humboldt University Berlin, which distributes the high-intensity computational processing to a server rather than the user’s own computer. After running the algorithm, the user can save the results to their computer, and/or view the formulas as a data layer in Constellation’s graphing area.

Secondly, two new algorithms for finding chemically meaningful mass defect units/bases for unsupervised trend detection in HRMS data were developed. After a library of potential units/bases with associated formulas is generated (based on certain user-adjustable limits), it is passed on to one of two unit/base finding algorithms, where each unit/base is evaluated in comparison with the raw HRMS dataset. Upon meeting certain requirements, units/bases are saved to a final output list. If “untargeted” mode is selected, this unit/base list is directly passed to the trend finding part of the software. If “targeted” mode is chosen, a unit/base selection box is displayed and populated, showing both the accurate mass and associated formula for each unit/base, and allowing the user to select which units/bases they would like to use in the trend search to follow. Both approaches were evaluated on several open-source HRMS datasets, and between the two unit/base finding algorithms, all previously identified changing units in the data were successfully identified. Here, “untargeted” mode demonstrated its utility as a good “starting point” for this evaluation, after which some customization in “targeted” mode was necessary to identify all the changing units in this analysis, due to the differing capabilities of algorithms I and II.

Finally, in migrating Constellation to our own servers and upgrading several interface components, we were able increase all file-size limitations to a maximum of 10 mb for both uploaded raw MS data and data generated from the formula finding or trend finding algorithms. This is a significant upgrade which will make the Constellation software environment more useful and accessible to a larger variety of users, especially given the large size of typical HRMS datasets, and enable further opportunities to develop new HRMS data-processing tools by borrowing from the expanded data structures, algorithms, and graphical interfaces that we have developed here. In general, there remain several unexplored possibilities in the realm of unsupervised data analysis for high-resolution mass spectrometry. We hope that future developments in this area, such as integrating additional tools from the open-source CoreMS software into Constellation (i.e., adding the ability to upload MS data in raw vendor formats), can continue to demonstrate how collaborative work in the open-source space can lead to multi-faceted solutions for mass spectrometry data processing, independent from the use of expensive and proprietary instrument manufacturer software.

## Availability and requirements

The open-source Constellation software described in this article can be accessed and used freely as a web application (no software downloads required) at the following website: https://constellation.chemie.hu-berlin.de.

- Project name: Constellation
- Project home page: https://scm.cms.hu-berlin.de/letournd/constellation
- Operating system(s): Platform independent web application
- Programming language: Python
- Other requirements: None
- License: GNU General Public License

## Data Availability

The “KendrickMassFilter_EvaluationDataset_PUBLIC” dataset used to evaluate the unit-finding algorithms is provided freely in the MassIVE Datasets database under the CC0 1.0 Universal license by the Center for Computational Mass Spectrometry, University of California, San Diego, USA (https://massive.ucsd.edu/ProteoSAFe/static/massive.jsp).
